# Supplementary material for: Glass Transition Behaviors of Poly (Vinyl Pyridine)/Poly (Vinyl Phenol) Revisited
Source: Polymers (Basel). 2019 Jul 5;11(7):1153. doi: 10.3390/polym11071153 (PMC6680477; doi:10.3390/polym11071153)
Supplement: Supplementary file 1 [file polymers-11-01153-s001.pdf]

Supporting Information for

Glass Transition Behaviors of Poly(vinyl pyridine) / Poly(vinyl phenol) Blends Revisited

Osamu Urakawa, and Ayaka Yasue*

Department of Macromolecular Science, Graduate School of Science, Osaka University

1-1 Machikaneyama-cho, Toyonaka, Osaka 560-0043, JAPAN

*Correspondence: Email: urakawa@chem.sci.osaka-u.ac.jp;

Tel/Fax: +81-6-6850-5538

(1) Detail of the differential scanning calorimetry data: determination of the glass transition temperatures.

Figure S1 shows the typical heat flow curves (denoted as DSC) in the heating and cooling scans for 2VPy40 / VPh16 blends with several compositions: blue and red lines represent the heating and cooling data, respectively. The time derivatives of the DSC curves are shown by the dashed lines denoted as DDSC. The glass transition temperatures (for both heating and cooling scans) were determined as the temperature at which the DDSC curve show a peak.

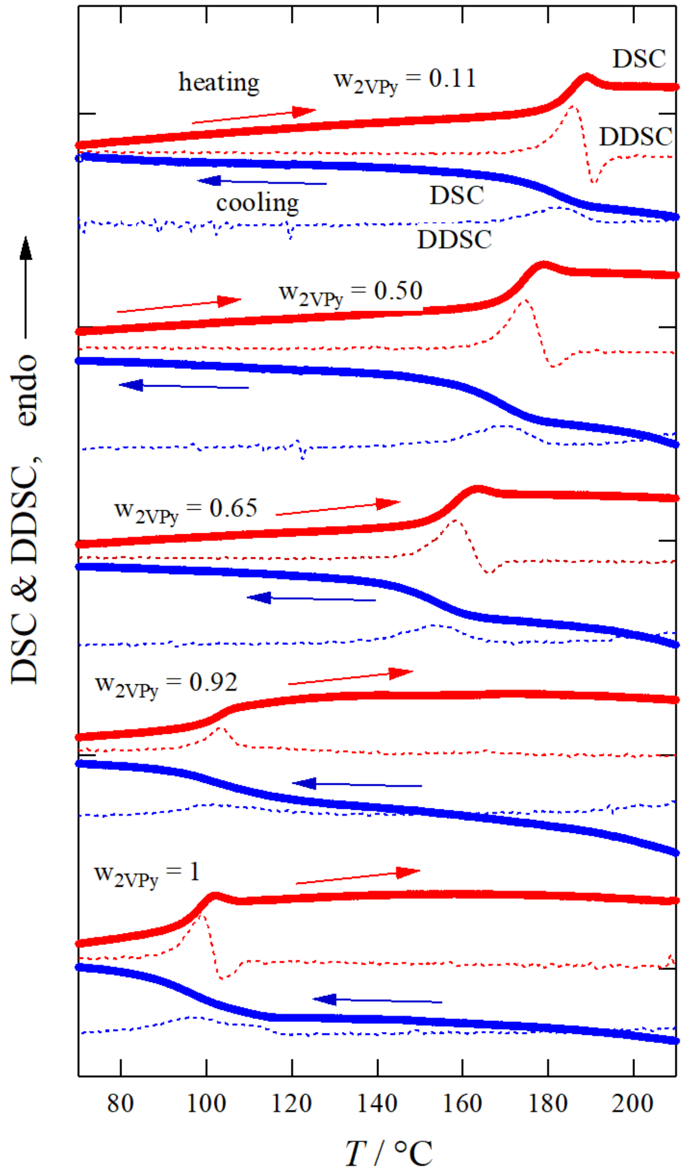

Figure S1 Heating and cooling scan DSC (heat flow) data for 2VPy40 / VPh16 blends with several compositions. The time derivatives of the DSC data denoted as DDSC are shown by dashed lines for both heating and cooling processes.

(2) DSC heating traces of 2VPy40 / VPh5, 2VPy40 / VPh2, and 4VPy60 / VPh5

Figure S2 shows the heating DSC traces (2nd run) for (a) 2VPy40 / VPh5, (b) 2VPy40 / VPh2, and (c) 4VPy60 / VPh5 blends with several compositions. All the blend samples were prepared by the method (II). The 3rd heating data were almost the same with these 2nd run data. T_g values were determined from the peak position of the time derivative of these DSC traces.

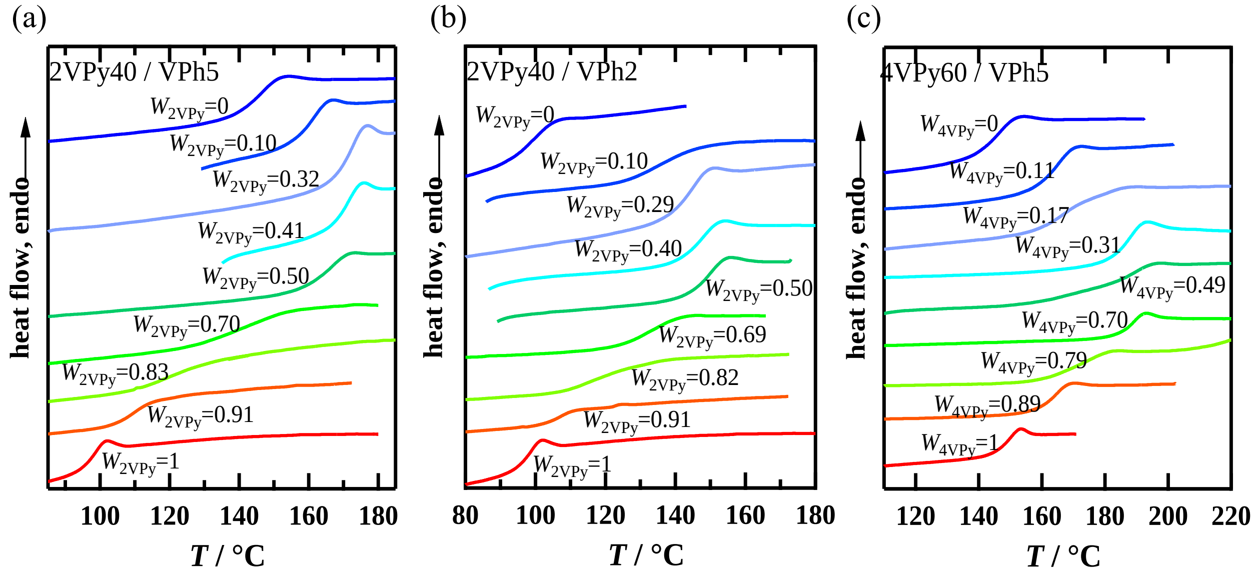

Figure S2 Heating DSC (heat flow) data for (a) 2VPy40 / VPh5, (b) 2VPy40 / VPh2, and (c) 4VPy60 / VPh5 blends with several compositions.

(3) Comparison of T_gs determined from the heating and cooling scans.

In Figures S3 (a), (b), (c), and (d), comparison of the heating T_g and cooling T_g data are shown for all the blends: (a) 2VPy40 / VPh16, (b) 2VPy40 / VPh5, (c) 2VPy40 / VPh2, and (d) 4VPy60 / VPh5. The data of heating T_gs are the same with those shown in Figures 4, 5, and 7 in the main text. In these figures, it is seen that weight fraction dependence of heating and cooling T_gs are similar while the absolute values are slightly different: heating T_g > cooling T_g. From these results, we can conclude that the effect of aging, which appeared as the enthalpy relaxation peaks in the DSC traces, does not seriously change the α values in the modified Kwei equation.

The bars in Figures S3 represent the width of the glass transition for the heating scan data. It was found that these widths were comparable with those observed in miscible polymer blends without specific interaction.[1]

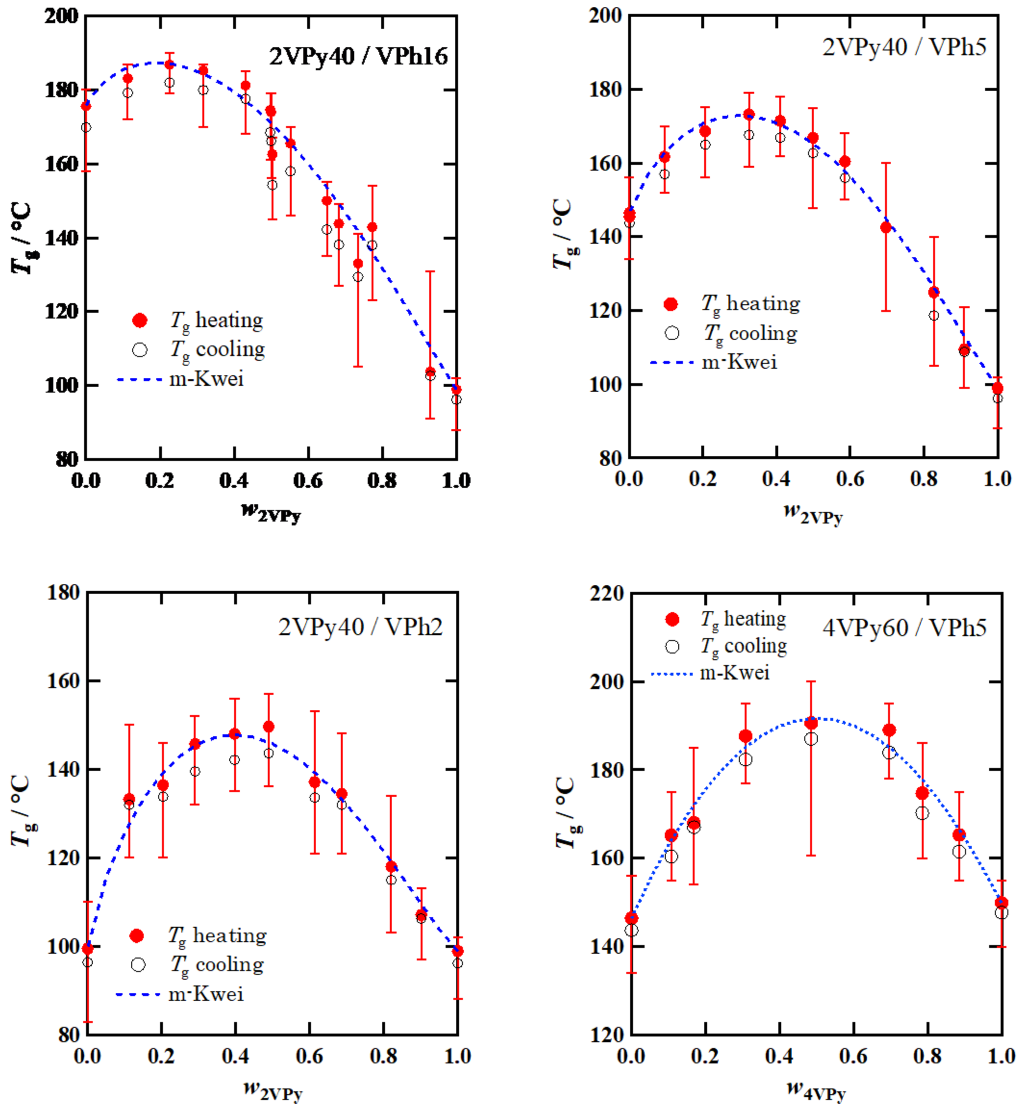

Figure S3 Comparison of the heating and cooling T_gs for the blends of (a) 2VPy40 / VPh16, (b) 2VPy40 / VPh5, (c) 2VPy40 / VPh2, and (d) 4VPy60 / VPh5. The vertical bars represent the width of DSC transition. The dashed lines are the fit-results with the modified Kwei equation.

Reference:

[1] Hirose, Y.; Urakawa, O.; Adachi, K. Dielectric study on the heterogeneous dynamics of miscible polyisoprene/poly(vinyl ethylene) blends: Estimation of the relevant length scales for the segmental relaxation dynamics. *Macromolecules* **2003**, *36*, 3699-3708, doi:Doi 10.1021/Ma0217940.
